# Intravenous Landiolol for Rate Control in Supraventricular Tachyarrhythmias in Patients with Left Ventricular Dysfunction: A Systematic Review and Meta-Analysis

**DOI:** 10.3390/jcm13061683

**Published:** 2024-03-14

**Authors:** Athina Nasoufidou, Andreas S. Papazoglou, Panagiotis Stachteas, Efstratios Karagiannidis, Athanasios Samaras, Sophia Alexiou, Michail-Angelos Mourtzos, George Kassimis, Nikolaos Fragakis

**Affiliations:** 1 Second Department of Cardiology, Aristotle University of Thessaloniki, 54124 Thessaloniki, Greece; athinanassi@gmail.com (A.N.); staxteasp@gmail.com (P.S.); stratoskarag@gmail.com (E.K.); asamaraa@auth.gr (A.S.); sophiealexiou@yahoo.com (S.A.); michaelmourtzos@gmail.com (M.-A.M.); gksup@yahoo.gr (G.K.); 2 Athens Naval Hospital, 11521 Athens, Greece; anpapazoglou@yahoo.com

**Keywords:** landiolol, heart failure, atrial fibrillation, atrial flutter, arrhythmia, tachycardia, supraventricular, ventricular dysfunction, beta-blocker, rate control

## Abstract

**Background:** This systematic review explores the effects of landiolol administration in individuals presenting with supraventricular tachyarrhythmia (SVT) and concurrent left ventricular dysfunction, without being septic or in a peri-operative period. **Methods:** We systematically searched PubMed, Cochrane, Web of Science, and Scopus databases, retrieving a total of 15 eligible studies according to prespecified eligibility criteria. **Results:** Patients treated with landiolol experienced a substantial reduction in heart rate (HR) (mean HR reduction: 42 bpm, 95% confidence intervals (CIs): 37–47, I^2^ = 82%) and were more likely to achieve the target HR compared to those receiving alternative antiarrhythmic therapy (pooled odds ratio (OR): 5.37, 95% CIs: 2.87–10.05, I^2^ = 0%). Adverse events, primarily hypotension, occurred in 14.7% of patients receiving landiolol, but no significant difference was observed between the landiolol and alternative antiarrhythmic receiving groups (pooled OR: 1.02, 95% CI: 0.57–1.83, I^2^ = 0%). No significant difference was observed between the two groups concerning sinus rhythm restoration (pooled OR: 0.97, 95% CI: 0.25–3.78, I^2^ = 0%) and drug discontinuation due to adverse events (pooled OR: 5.09, 95% CI: 0.6–43.38, I^2^ = 0%). **Conclusion:** While further research is warranted, this systematic review highlights the potential benefits of landiolol administration in the management of SVTs in the context of left ventricular dysfunction.

## 1. Introduction

Heart failure (HF) and supraventricular tachyarrhythmias (SVTs) frequently coexist. Their concurrence aggravates the clinical presentation of each individual condition, further impairing the underlying cardiac function and hemodynamic status. Atrial fibrillation (AF), the most common atrial tachyarrhythmia in HF patients, results in a shortened diastolic filling period of the left ventricle, lack of atrial contraction, and tachycardia-induced cardiomyopathy, while HF causes atrial dilatation, as a consequence of elevated atrial pressures, and atrial fibrosis [1]. Patients with chronic HF, who develop AF, have a greater risk of cardiovascular death or hospitalization, compared to those who preserve sinus rhythm (SR) [2]. Moreover, tachycardia-induced cardiomyopathy seems to have detrimental outcomes compared to other etiologies of HF [3].

In the clinical setting of hemodynamic instability due to AF, rhythm control is the preferred management strategy. Otherwise, a rate control strategy can be considered as an initial approach prior to confirming adequate coagulation status or planning further management. European Society of Cardiology guidelines validate the use of beta-blockers for rate control in HF with reduced ejection fraction (HFrEF) (Class of recommendation I, Level of evidence A) [4] and mildly reduced ejection fraction (HFmEF) (Class of recommendation IIa, Level of evidence B) [5], due to their established safety. Digoxin can be used, supplementary to beta-blockers when the ventricular rate remains high, or when beta-blockers are contraindicated or not tolerated (Class of recommendation IIa, Level of evidence C) [4]. Adequate rate control is considered to be a heart rate (HR) ≤110 bpm at rest as a primary strategy, unless symptoms or LV dysfunction persist or biventricular pacing cannot be achieved in CRT. In that instance, a lower targeting HR (<80 bpm) is ideal, as well as restoration of SR.

Landiolol is an ultra-short-acting beta-1 blocker with a half-life of approximately 4 min [6]. Landiolol displays a remarkable high cardiac β1 selectivity (β1/β2 selectivity ≈ 251) [7] compared to other intravenous beta-blockers (β1/β2 selectivity ≈ 33 for esmolol and 2.3 for metoprolol) [8]. Because of these properties, landiolol can lower HR without blood pressure decreasing alongside. Moreover, in the need of discontinuation of treatment, landiolol administration can be rapidly ceased and quickly initiated again [7]. Landiolol has been used for rate control management in several critical conditions such as post-operatively [9], in intensive care units [10], in critically ill COVID-19 patients [11], in sepsis [12], septic shock [10], and in acute decompensated HF, with satisfying results. This systematic review aims to investigate the effect of landiolol administration in non-septic or operated patients who present with SVT and have comorbid left ventricular dysfunction.

## 2. Materials and Methods

### 2.1. Search Strategy—Study Selection

The study was prospectively registered in the PROSPERO database (PROSPERO 2023 CRD42023448712). Our meta-analysis is reported according to the Preferred Reporting Items for Systematic Reviews and Meta-Analyses (PRISMA) checklist [13] (Supplementary Table S1). A systematic electronic search of published research was conducted using the PubMed, Cochrane, Web of Science, and Scopus databases from database inception up to 14 July 2023. The search included solely the term: “landiolol” to avoid missing any relevant study. The reference lists of the included studies and relevant reviews were also hand-searched to identify further relevant studies. 

### 2.2. Inclusion Criteria

Any observational cohort studies or randomized trials were included if they reported the effects of landiolol treatment on HR/blood pressure/adverse events/follow-up outcomes among adult individuals (>18 years old) presenting with SVT and comorbid left ventricular dysfunction, without being septic or in a peri-operative period. No restrictions in terms of race or ethnicity were adopted. Two investigators (A.N. and A.S.P.) independently examined all titles and abstracts and obtained full texts of potentially relevant papers. Working independently, we read the papers and determined whether they met the inclusion criteria. Discrepancies were resolved by consensus, referring back to the original article, in consultation with a third author (E.K.). 

### 2.3. Exclusion Criteria

Exclusion criteria of the meta-analysis were the following: (1) case reports or case series with less than 10 patients, reviews, editorials, and practice guidelines; (2) studies conducted in pediatric populations; and (3) articles published in non-English language.

### 2.4. Data Extraction

For all eligible studies, we extracted information on study design, study size, source of data, population characteristics, duration of follow-up, outcomes of interest, and matching and confounding factors. Specifically, we recorded for each study the following characteristics according to the drug delivered (where available): n (%) of patients (i) achieving target HR, (ii) restoring to SR, (iii) reporting adverse events or subjective symptoms (leading to drug discontinuation), (iv) with comorbid diabetes mellitus, hypertension, valvular heart disease, and coronary artery disease. We also recorded the New York Heart Association (NYHA) classification, prior medication (beta-blockers, diuretics, angiotensin-converting enzyme inhibitors (ACEi)/angiotensin receptor blockers (ARBs), mineralocorticoid receptor antagonists (MRAs), amiodarone, and digitalis), the percentage reduction in HR, the percentage reduction in systolic and diastolic blood pressure, the male percentage, the mean age, and the mean values of pre- and post-treatment: brain natriuretic peptide (BNP), left ventricular ejection fraction (LVEF), left ventricular end-diastolic diameter (LVEDd), left ventricular end-systolic diameter (LVESd), left atrial diameter (LAd), pulmonary capillary wedge pressure (PCWP), systemic vascular resistance (SVR), cardiac index (CI), and glomerular filtration rate (GFR)/creatinine, where available.

### 2.5. Quality and Risk of Bias Assessment

The quality of the included cohort studies was assessed using the Newcastle–Ottawa Scale (NOS) [14], as recommended by the Cochrane Collaboration. This scale uses a star system to assess the quality of a study in three domains: selection, comparability, and outcome. Randomized control trials were evaluated using the Risk of Bias tool of the Cochrane Collaboration [15].

### 2.6. Outcomes of Interest

Primary outcome of interest was the target HR achievement, which was defined as an at least 20% reduction from the initial HR with the final HR being less than 110 bpm. SR restoration and reporting of adverse events or subjective symptoms leading to drug discontinuation were the secondary outcomes of interest in the present meta-analysis. 

### 2.7. Statistical Analysis—Data Synthesis

Rates of target HR achievement, SR restoration, and adverse events have been recorded for both case (patients receiving landiolol treatment) and control (patients receiving non-landiolol treatment) groups. For the categorical outcomes of interest (target HR achievement, SR restoring, and adverse events report), data synthesis was conducted through dichotomous outcome random effects meta-analysis (with Mantel–Haenszel weighting and the DerSimonian–Laird method) producing pooled odds ratios (ORs) with 95% confidence intervals (CIs), depending on the administration of landiolol or non-landiolol drug.

For the continuous outcomes of interest (pre- and post-treatment blood pressure, HR and LVEF values), a random effects meta-analysis was performed to pool their mean difference pre- and post-landiolol administration. Forest plots were constructed to show the overall effect of landiolol administration in each parameter. 

The observed heterogeneity in each analysis was described using the I^2^ statistic, which was quantified as low (<25%), moderate (25% to 75%), or high (>75%) heterogeneity [16]. Meta-regression analysis could not be performed because of the limited number of eligible studies. The possibility of publication bias was visually evaluated after generating funnel plots; the Egger’s test could not be performed because of the limited number of eligible studies [17]. All statistical analyses were performed using Review Manager (RevMan), Version 5.4., 2020, with 2-tailed *p*-values of less than 0.05 indicating statistical significance.

## 3. Results

**Figure 1:**
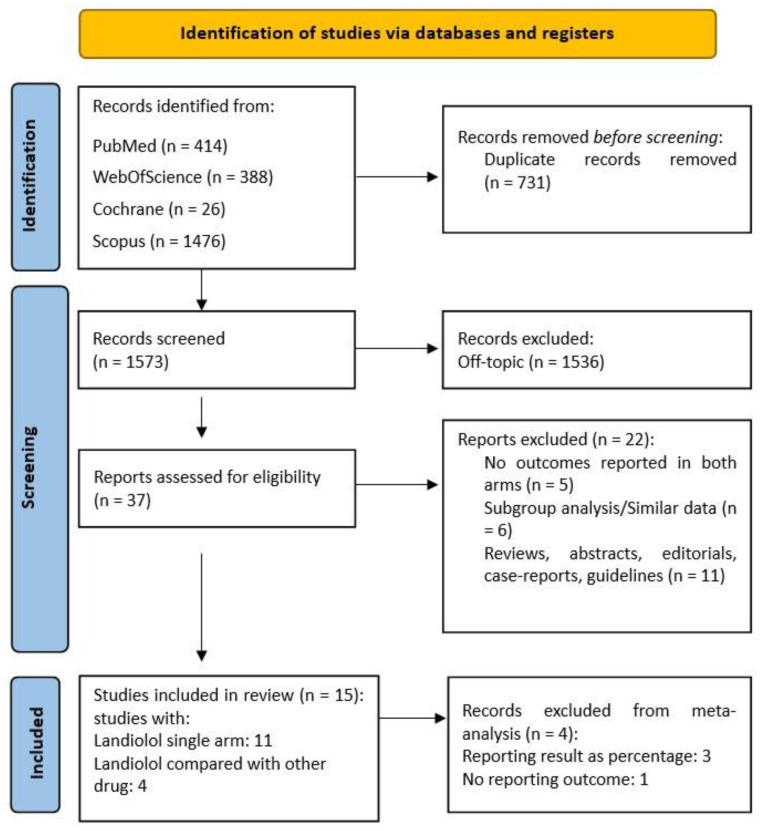
PRISMA 2020 flow diagram.

A total of 2304 articles were initially retrieved. Ultimately, 15 studies satisfied the predefined eligibility criteria for the systematic review and 11 studies were included in the meta-analysis. In four studies, landiolol was compared to other antiarrhythmic therapy (in two studies with digoxin and in two studies with diltiazem), while seven studies were single-arm. The detailed flow diagram of the study selection process is presented in Figure 1. 

### 3.1. Quality Assessment and Publication Bias

The quality assessment of the included studies showed an overall good quality with low risk of bias in all of them (Supplementary Figure S1).

### 3.2. Study Characteristics and Data Synthesis

The patient sample size added up to a total of 1674 patients. The design and main characteristics of the selected studies are presented in Table 1. Among the studies included, one is a randomized controlled trial, while the remaining studies are observational. Baseline patient characteristics are presented in Table 2.

**Figure 2:**
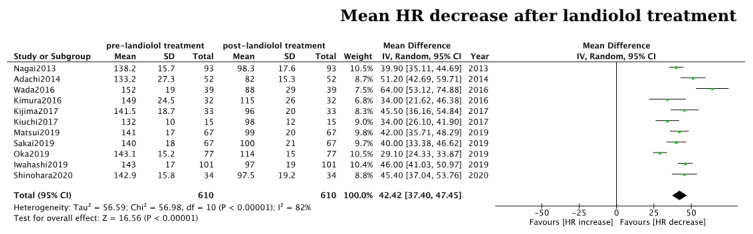
Forest plot of the mean heart rate reduction achieved with landiolol administration. Green dots represent the mean HR decrease of each study. The black diamond represents the overall mean HR reduction from all included studies [18,19,20,21,24,26,27,28,29,32,33].

**Figure 3:**
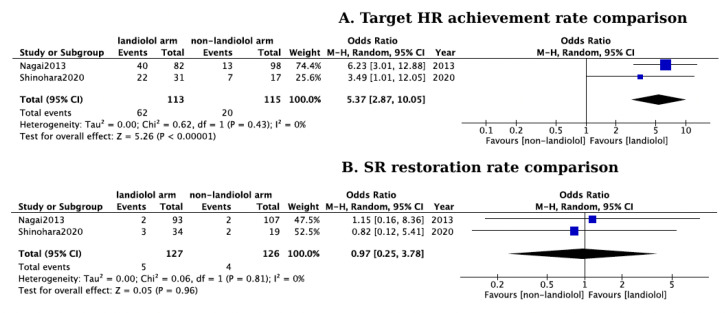
(**A**) Forest plot of comparison in target HR achievement rates between landiolol and non-landiolol treated groups Blue squares represent the mean Odds ratio for target HR achievement of each study and the black diamond represents the overall pooled Odds ratio for target HR achievement from all the included studies [18,19]; (**B**) Forest plot of comparison in sinus rhythm restoring rates between landiolol and non-landiolol treated groups. Blue squares represent the mean Odds ratio for SR restoration of each study and the black diamond represents the overall pooled Odds ratio for SR restoration from the included studies [18,19].

Patients treated with landiolol exhibited a notable HR decrease (mean HR reduction: 42 bpm, CI: 37–47, *p* < 0.01, I^2^ = 82%; Figure 2), with 75% of the treated patients successfully reaching the target HR. In comparison to alternative antiarrhythmic treatments like digoxin and diltiazem, landiolol exhibited superior effectiveness in regulating HR (pooled OR: 5.37, 95% CI: 2.87–10.05, *p* < 0.01, I^2^ = 0%; Figure 3A), as evidenced by three out of the four eligible studies [18,21,34]. In the fourth study, both landiolol and diltiazem exhibited comparable efficacy in reducing HR. However, a notable safety difference emerged in that study as diltiazem significantly lowered blood pressure, an adverse event not observed in the landiolol group [20]. 

Among the included studies, only two of them provided data on SR restoration, and no statistically significant difference was observed among landiolol- and non-landiolol treated patients (pooled OR: 0.97, 95% CI: 0.25–3.78, *p* > 0.05, I^2^ = 0%; Figure 3B).

**Figure 4:**
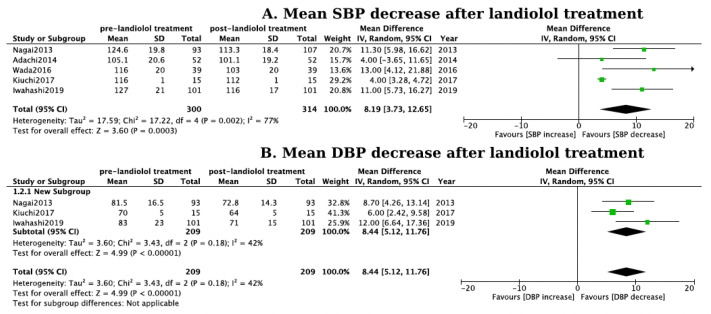
(**A**) Forest plot of the systolic blood pressure reduction after landiolol treatment. Green squares represent the mean SBP decrease of each study. The black diamond represents the overall mean SBP decrease from the included studies [18,20,24,26,27]; (**B**) Forest plot of the diastolic blood pressure reduction after landiolol treatment. Green squares represent the mean DBP decrease of each study. The black diamond represents the overall mean DBP decrease from the included studies [18,20,26].

**Figure 5:**
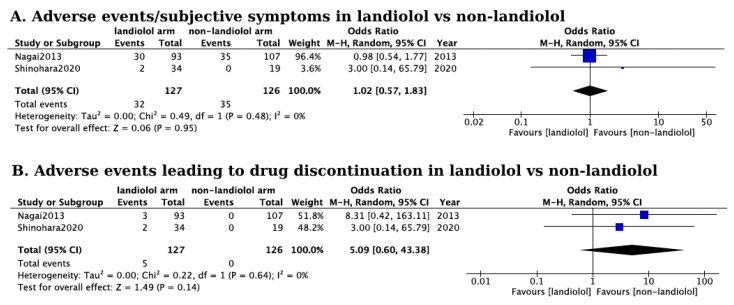
(**A**) Forest plot of comparison in the rates of adverse events or subjective symptoms report in landiolol and non-landiolol treated groups. The blue squares represent the Odds ratio of adverse events/subjective symptoms from each study. The black diamond represents the overall pooled Odds ratio of adverse events/subjective symptoms from the included studies [18,19]; (**B**) Forest plot of comparison in the rates of adverse events leading to drug discontinuation in landiolol and non-landiolol treated groups. The blue squares represent the Odds ratio of adverse events leading to drug discontinuation of each study. The black diamond represents the overall pooled Odds ratio of adverse events leading to drug discontinuation from all included studies [18,19].

Regarding safety, 14.7% of the patients treated with landiolol reported an adverse event or subjective symptoms, most frequently characterized by a dose-dependent reduction in systolic and diastolic blood pressure (mean systolic blood pressure decrease by 8.19 mmHg, CI: 3.73–12.65 mmHg, Figure 4A and mean diastolic pressure decrease by 8.44 mmHg, CI: 5.12–11.76 mmHg, Figure 4B). However, drug discontinuation occurred in only 6% of the patients treated with landiolol. The blood pressure cut-off points for discontinuing drug administration were set at <80 mmHg [19,24,27,29,31] or <90 mmHg [18,26,28]. Patients with a systolic blood pressure (SBP) below these thresholds upon presentation were excluded from the studies. Cardiogenic shock is clearly mentioned to have manifested in four patients, with the subsequent management remaining unclear. No significant difference was observed in the rates of adverse events or subjective symptom report between patients receiving landiolol and those receiving other antiarrhythmic therapies (pooled OR: 1.02, 95% CI: 0.57–1.83, *p* > 0.05, I^2^ = 0%; Figure 5A). Also, no significant difference was observed in the rates of adverse events leading to drug discontinuation (pooled OR: 5.09, 95% CI: 0.6–43.38, *p* > 0.05, I^2^ = 0%; Figure 5B).

## 4. Discussion

In our meta-analysis, landiolol treatment led to significant HR reduction (mean reduction = 42 (95% CI: 37–47) bpm) and to target HR achievement in 75% of the treated patients presenting with SVT and concurrent left ventricular dysfunction. Despite the limited number of two-armed studies, landiolol showed superior effectiveness in target HR achievement but not in SR restoration when compared to alternative medications (digoxin and diltiazem). Landiolol demonstrated good tolerability, with a minimal percentage (6%) of patients necessitating drug discontinuation, typically attributed to hypotension; however, no significant difference was observed between landiolol and non-landiolol treated groups. 

For patients with HF who experience tachyarrhythmia, the options for acute pharmaceutical interventions are limited [34]. For hemodynamically stable patients, opting for rate control is a viable strategy until further planning is established, as in cases of AF, when ensuring the coagulation status precedes cardioversion [35,36,37]. In patients with LVEF < 40%, apart from beta-blockers, only digoxin is an alternative option for rapid rate control [38]. However, digoxin requires vigilant monitoring and has a half-life of about 36–44 h, which may be prolonged in renal dysfunction and older age [39]. On the other hand, beta-blockers can achieve HR control, but their efficacy depends on their beta-1 selectivity, as highlighted in a recent meta-analysis [40]. This analysis by Perrett et al. revealed that only patients treated with landiolol achieved the target HR. Moreover, the findings indicated that non-selective beta-blockers and beta-1 selective blockers resulted in a higher incidence of side effects such as hypotension and bradycardia, in contrast to landiolol, which was characterized as a beta-1 super selective agent [40]. Furthermore, within the included studies in our meta-analysis, diltiazem was used as a comparable drug to landiolol. Diltiazem is a well-considered pharmaceutical option but has the limitation of being indicated only in patients with preserved LVEF, preserved exercise capacity, and low natriuretic peptides [41]. Moreover, it is noteworthy that there is a lack of clinical trials directly comparing landiolol to amiodarone, which is regarded as the ultimate recourse when heart rate is irrepressible. Amiodarone can be used for rate control in HFmEF and HFrEF [42], but it is only indicated in combination therapy and in absence of thyroid disease [43,44].

An additional advantage of landiolol and beta-blockers in general (amiodarone is also a β-adrenergic receptor (β-AR) antagonist) lies in their modulation of the sympathetic nervous system. The role of the autonomic nervous system in both the initiation and perpetuation of arrhythmias is well-established [45]. Atrial remodeling and shortening of atrial refractory period due to AF is also confirmed [46,47]. B-AR blockade decreases the rapid-delayed rectifier K current (I_Kr_) and slow-delayed rectifier K current (I_Ks_) and enhances the L-type Ca current (I_CaL_), resulting in the prolongation of action potential duration [48,49]. Thus, beta-blockade inhibits sympathetic hyperactivity often associated with AF with rapid ventricular response [46]. Patients undergoing chronic BB therapy also experience a phenomenon known as upregulation, signifying an increase in adrenoceptor density when cells are exposed to BBs. This mechanism enhances the receptors’ responsiveness to sympathetic stimulation and benefits only patients with chronic HF receiving BBs [50]. BB-naïve patients may have a more unpredictable hemodynamic response or HR reduction and the initiation of a BB should be at the lower dose with a cautious approach to subsequent uptitration [34].

Landiolol has also been used in acute decompensated HF treated with inotropes (levosimendan) [51,52]. The minimal negative inotropic impact of landiolol, coupled with its pronounced negative chronotropic effect, enabled the de-escalation of catecholamine dosages and was well-tolerated. In a case series involving eleven critically ill patients with acute decompensated heart failure (ADHF) due to tachyarrhythmia, the co-administration of landiolol with levosimendan or norepinephrine resulted in gradual hourly improvements, including increased systolic blood pressure (SBP), reduced heart rate (HR), and successful restoration of sinus rhythm in over 80% of patients [53]. Landiolol has been used in combination with inotropes like dobutamine and noradrenaline in several studies, delivering favorable outcomes [54,55]. In a number of studies, landiolol was co-administered with milrinone, resulting in increased stroke volume index and significant decrease in pulmonary capillary wedge pressure (PCWP) [56,57,58]. In scarce cases, landiolol has been also used in patients with severe hypotension and advanced HF [54]. Hence there is a substantial body of observational data supporting the safety and efficacy of landiolol administration. However, further randomized data are expected prior to reaching definite conclusions regarding the superiority of landiolol over other antiarrhythmic drugs in HF patients with SVT.

Apart from SVTs, landiolol has been examined in ventricular arrhythmias when patients did not respond to class III anti-arrhythmic drugs with satisfying inhibition rates of recurrent unstable ventricular tachycardia, recurrent ventricular fibrillation, and electrical storm [59,60]. 

Regarding the prevalent respiratory side effects associated with BBs, landiolol is typically contraindicated in cases of acute asthmatic attacks. In a literature review focusing on individuals with pre-existing asthma, the use of cardio-selective beta-1 blockers was not associated with increased exacerbations of asthma [61]. However, BBs should be used with caution in these patients.

In summary, landiolol demonstrates highly promising and positive outcomes in the management of tachyarrhythmias across diverse clinical scenarios such as ADHF, sepsis, and critically ill patients, whether used as a standalone therapy or in combination with other anti-arrhythmic drugs. For the widespread utilization of landiolol, the conduction of extensive large-scale RCTs with diverse ethnic groups is required. Hospitalized patients with heart failure and tachyarrhythmia experience considerable clinical burden, making the attainment of clinically significant results of any treatment challenging.

## 5. Limitations

The main limitation of our meta-analysis is the limited number of eligible studies and the lack of randomized controlled trials. The absence of a control group in single-arm studies further restricts the power of the performed between-drug comparisons. Additionally, most studies feature a small number of participants and have differences in data presentation (e.g., some studies report the absolute HR reduction, while others only the HR reduction percentage), which could not be resolved through contact with corresponding study authors and made the appropriate data synthesis even more challenging.

## 6. Conclusions

In conclusion, the efficacy of landiolol in reducing HR appears to be consistent among patients with HF experiencing an SVT. Landiolol exhibits a pronounced negative chronotropic effect, with a minimal, dose-dependent, and rapidly reversed negative inotropic impact. Further randomized data are warranted to conclusively demonstrate the effectiveness and safety of landiolol in this clinical setting. 

## Figures and Tables

**Table 1:** Characteristics of the included studies.

Study ID	Type of Study	Population (Main Characteristics)	Total N, Landiolol Dosage/Comparator	Follow-Up	Main Outcomes
Nagai et al. 2013 [18]	Multicenter, single-blind, RCT (J-Land Study)	🟀 Age ≥ 20 years 🟀 NYHA class III/IV 🟀 AF/AFL 🟀 HR ≥ 120 bpm 🟀 LVEF: 25–50%	214 patients Landiolol (n = 99) Digoxin (n = 115) 1–10 μg/kg/min ≥2 h and up to 72 h	-	🟀 HR was significantly lower in the landiolol group than in the digoxin group at 1 h (117.3 vs. 125.4 bpm) and 2 h (110.2 vs. 122.3 bpm) 🟀 The change in HR from baseline to 2 h was −27.0 ± 13.3 bpm in the landiolol group and −16.0 ± 13.0 bpm in the digoxin group 🟀 Changes in systolic and diastolic BP over time were not significantly different between the 2 groups
Shinohara et al. 2020 [19]	Single-center retrospective observational study	🟀 AF 🟀 LVEF ≤ 25%	53 patients Landiolol (n = 34) Digoxin (n = 19) 0.5–10 μg/kg/min for 24 h	-	🟀 Landiolol treatment was significantly associated with responders at 24 h 🟀 2 patients in the landiolol group experienced hypotension, which recovered immediately after discontinuation of treatment
Kiuchi et al. 2017 [20]	Single-center retrospective observational study	🟀 Acute HF complicated by SVT 🟀 EF < 50%	59 patients Diltiazem (n = 44) Landiolol (n = 15) Maximum dose of landiolol: 5.57 ± 4.78 μg/kg/min	-	🟀 Both landiolol and diltiazem reduced HR 🟀 Target HR was achieved more frequently in the landiolol group 🟀 SBP and DBP were not decreased in landiolol group, but significantly decreased in diltiazem group
Kimura et al. 2016 [21]	Single-center prospective observational study	🟀 HF patients (mean EF: 30% and 36% in the two groups) 🟀 SVT (AF, AFL, Atrial tachycardia) 🟀 HR > 110 bpm	64 patients Landiolol (n = 32) Diltiazem (n = 32)	-	🟀 HR reduction: 23.1 ± 17.8% in the landiolol group and 14.5 ± 15.7% in the diltiazem group (*p* = 0.044) 🟀 BP reduction: 7.5 ± 22 mmHg and 2.3 ± 17 mmHg, not statistically significant
Yamashita et al. 2019 [22]	Multicenter prospective observational study (AF-CHF landiolol survey) Single-arm	🟀 NYHA class III/IV 🟀 LVEF: 25–50% 🟀 AF/AFL with HR ≥ 120 bpm	1121 patients Highest rate 4.1 ± 3.3 μg/kg/min for 80.9 ± 100.9 h	6 months	🟀 Mean heart rate decreased substantially after treatment with landiolol by >20% in 77.5% of patients. 🟀 174 adverse events occurred in 140 patients (12.5%) 🟀 6-month all-cause mortality rate was 14.6% 🟀 Male sex and advanced age were independently associated with all-cause mortality and death from HF [23]
Adachi et al. 2014 [24]	Single-center prospective observational study Single-arm	🟀 SVT with HR ≥ 100 bpm 🟀 Congestive HF 🟀 LVEF < 40 % 🟀 Tachycardia for ≥ 1 h after standard treatment	52 patients Average dose: 10.8 ± 9.4 μg/kg/min for 3 ± 1 days	-	🟀 Reduction in heart rate from 133.2 ± 27.3 bpm at baseline to 82.0 ± 15.3 bpm after administration of landiolol 🟀 SBP remained unchanged 🟀 Improvement in EF, from 32.3 ± 11.9% at baseline to 39.7 ± 6.5% after rate control 🟀 In 3 patients, landiolol administration was ceased due to hypotension
Shirotani et al. 2022 [25]	Single-center prospective case-control	🟀 ADHF 🟀 AF 🟀 HR ≥ 130 bpm at admission	60 patients Landiolol group (n = 37) Reference group (n = 23)	24 h	🟀 HR was lower in the landiolol group (111.6 vs. 97.9 bpm, *p* = 0.02) 🟀 Absolute HR reduction was greater in the landiolol group (−32.2 vs. −50.0 bpm, *p* = 0.006) 🟀 Lower all-cause mortality in the landiolol group (adjusted hazard ratio: 0.15, 95% CI: 0.02–0.92)
Iwahashi et al. 2019 [26]	Single-center prospective observational study Single-arm	🟀 Inpatients ≥20 years old 🟀 NYHA class IV 🟀 AF with an LVEF < 40% 🟀 HR > 120 bpm	101 patients Average dose: 3.8 ± 2.3 μg/kg/min for 24 h	30 days	🟀 94% achieved target HR in ≤24 h (defined as <110 bpm with a 20% decrease in basal HR) 🟀 No serious side effects were reported 🟀 Landiolol administration resulted in improved cardiac ultrasound measurements 🟀 Death due to HF 3 (3%) 🟀 HF prolongation 14 (13.8%)
Wada et al. 2016 [27]	Single-center retrospective observational study Single-arm	🟀 Rapid AF, AFL, atrial tachycardia with ventricular response ≥ 120 bpm 🟀 LVEF < 50%	39 patients 1–10 μg/min/kg, until a positive/adverse effect appeared Infusion: 3 (1–7) days	-	🟀 HR reduction from 152 ± 19 bpm to 88 ± 29 bpm 🟀 EF < 25% was identified as the only independent factor of non-responders to treatment 🟀 54% conversion to sinus rhythm 🟀 3 patients experienced an adverse event 🟀 Survival 79%
Matsui et al. 2019 [28]	Single-center retrospective observational study Single-arm	🟀 AF, aflutter, and fast atrial tachyarrhythmia 🟀 ADHF (clinical diagnosis at first)	67 patients 1–12 μg/kg/min for 5 (1–24) days	16 ± 12 months	🟀 Landiolol reduced HR 141 ± 17 bpm at baseline to 99 ± 20 bpm at 6 h (*p* < 0.001) 🟀 No remarked reduction in blood pressure or deterioration of HF 🟀 15 (22%) patients restored sinus rhythm 🟀 Patients with sinus rhythm had a lower frequency of rehospitalization due to worsening HF than patients with ATAs
Oka et al. 2019 [29]	Single-center retrospective observational study Single-arm	🟀 NYHA class III or IV 🟀 LVEF < 50% 🟀 HR ≥ 120 bpm 🟀 AF/AFL/atrial tachycardia	77 patients AF (n = 65) AFL/atrial tachycardia (n = 12) 1–10 μg/kg/min for 24 h or until HR < 110 bpm	-	🟀 Landiolol significantly decreased HR in all groups 🟀 Greater HR reduction in the AF group 🟀 HR percent decrease from baseline to 12 h was −10.2 ± 12.7% in the AFl/atrial tachycardia group and −28.3 ± 13.2% in the AF group (*p* < 0.001) 🟀 In 10 patients, landiolol discontinuation due to hypotension (9) and bradycardia (1) 🟀 HR reduction 30% in the AF group and 10% in the AFL/atrial tachycardia group
Kobayashi et al. 2012 [30]	Single-center prospective observational study Single-arm	🟀 ADHF with LVEF ≤ 0.35 🟀 HR > 90 bpm 🟀 Tachycardia for ≥4 h after standard treatment	20 patients 1.5–6.0 μg/kg/min for 5 ± 2 days	-	🟀 Dose-dependent HR reduction 🟀 Improved hemodynamic parameters as pulmonary capillary wedge pressure 🟀 No adverse effects were recorded
Kobayashi et al. 2014 [31]	Single-center prospective observational study Single-arm	🟀 ADHF 🟀 With rapid AF (HR ≥ 120 bpm) 🟀 NYHA class III–IV and systolic/diastolic HF	23 patients Systolic heart failure (n = 12), diastolic heart failure (n = 11) 1–2 μg/kg/min for 24 h	-	🟀 HR reduced by 22% in 2 h after starting landiolol (baseline: 142.8 ± 18.4 bpm) 🟀 No incidence of hypotension was recorded
Kijima et al. 2017 [32]	Single-center prospective observational study Single-arm	🟀 Acute HF 🟀 AF, aflutter, atrial tachycardia 🟀 LV dysfunction	33 patients Landiolol monotherapy vs. landiolol + dobutamine	-	🟀 Heart rate decreased by 31.5 ± 13.8% in the landiolol monotherapy group and 27.1 ± 11.7% on the landiolol + dobutamine group 🟀 BP reduction was equal in both groups
Sakai et al. 2019 [33]	Single-center prospective observational study Single-arm	🟀 ADHF 🟀 AF or atrial tachycardia	67 patients 50 matched controls 3.0 (1.0–12.0) μg/kg/min for 5 (1–24) days	16 ± 12 months	🟀 Mean HR decreased significantly from 140 ± 18 to 100 ± 21 bpm (*p* < 0.05) 🟀 No difference was recorded in BP 🟀 Restoration of sinus rhythm in 15 (22%) patients 🟀 Higher rate of death or HF rehospitalization in patients without restoration of sinus rhythm

RCT: randomized control trial, ΝΥHA class: New York Heart Association Functional Classification, AF: atrial fibrillation, AFL: atrial flutter, ADHF: acute decompensated heart failure, HF: heart failure, SVT: supraventricular tachycardia, LVEF: left ventricle ejection fraction, bpm: beats per minute, ΒP: blood pressure, SBP: systolic blood pressure, DBP: diastolic blood pressure, h: hours, μg/kg/min: microgram per kilogram per minute.

**Table 2:** Baseline patient characteristics.

Baseline Characteristic	n/N (%)	Number of Studies with Available Data	Prior to Treatment	n/N (%)	Number of Studies with Available Data
**Male gender**	Landiolol: 58.8%	12/15	**Oral BBs**	196/506 (38.7%)	9/15
	Non-landiolol: 50.5%	4/5	**Diuretics**	157/299 (52.5%)	6/15
**Mean age (years)**	Landiolol: 64.8 ±15	11/15	**ACEi/ARB**	129/405 (31.9%)	8/15
	Non-landiolol: 73.3 ± 12.2	3/15	**MRA**	64/328 (19.5%)	7/15
**Diabetes Mellitus**	356/1331 (26.7%)	5/15	**Amiodarone**	47/181 (26%)	4/15
**Hypertension**	193/303 (63.7%)	5/15	**Digitalis**	34/353 (9.6%)	7/15
**Prior CAD**	1047/1627 (64.4%)	10/15	**Mean LVEF**	26.8 ± 19%	11/15
**Non-ischemic cardiomyopathy**	173/467 (37%)	8/15	**Mean BNP**	540.4 ± 830	10/15
**Valvular heart disease**	53/269 (19.7%)	5/15	**Mean HR (bpm)**	136.4 ± 23.4	14/15
**NYHA III/IV**	1037/1392 (74.5%)	6/15	**Mean SBP (mmHg)**	118.6 ± 25.2	10/15

CAD: coronary artery disease BB: beta-blocker, ACEi: angiotensin-converting enzyme inhibitors, ARB: angiotensin receptor blocker, MRA: mineralocorticoid receptor antagonists, LVEF: left ventricle ejection fraction, BNP: brain natriuretic peptide, HR: heart rate, SPB: systolic blood pressure.

## Data Availability

Our study data are available from the corresponding study author (N.F.) upon reasonable request.

## Supplementary Materials

The following supporting information can be downloaded at: https://www.mdpi.com/article/10.3390/jcm13061683/s1, Supplementary Figure S1: Quality assessment of the included studies with the Newcastle–Ottawa Scale and the Risk of Bias tool; Supplementary Table S1: PRISMA checklist as observed in the present meta-analysis.
