# Supplementary material for: Intravenous Landiolol for Rate Control in Supraventricular Tachyarrhythmias in Patients with Left Ventricular Dysfunction: A Systematic Review and Meta-Analysis
Source: J Clin Med. 2024 Mar 14;13(6):1683. doi: 10.3390/jcm13061683 (PMC10971001; doi:10.3390/jcm13061683)
Supplement: Supplementary file 1 [file jcm-13-01683-s001.zip › Search strategy_ Suppl.pdf]

Table S2. Detailed search strategy per database

Database	Documents
**PubMed**	"landiolol"[Supplementary Concept] OR "landiolol"[All Fields]
**Web of Science**	landiolol (All Fields)
**Cochrane**	(landiolol)
**Scopus**	ALL (landiolol)
