# Supplementary figures and images for: Intravenous Landiolol for Rate Control in Supraventricular Tachyarrhythmias in Patients with Left Ventricular Dysfunction: A Systematic Review and Meta-Analysis
Source: J Clin Med. 2024 Mar 14;13(6):1683. doi: 10.3390/jcm13061683 (PMC10971001; doi:10.3390/jcm13061683)

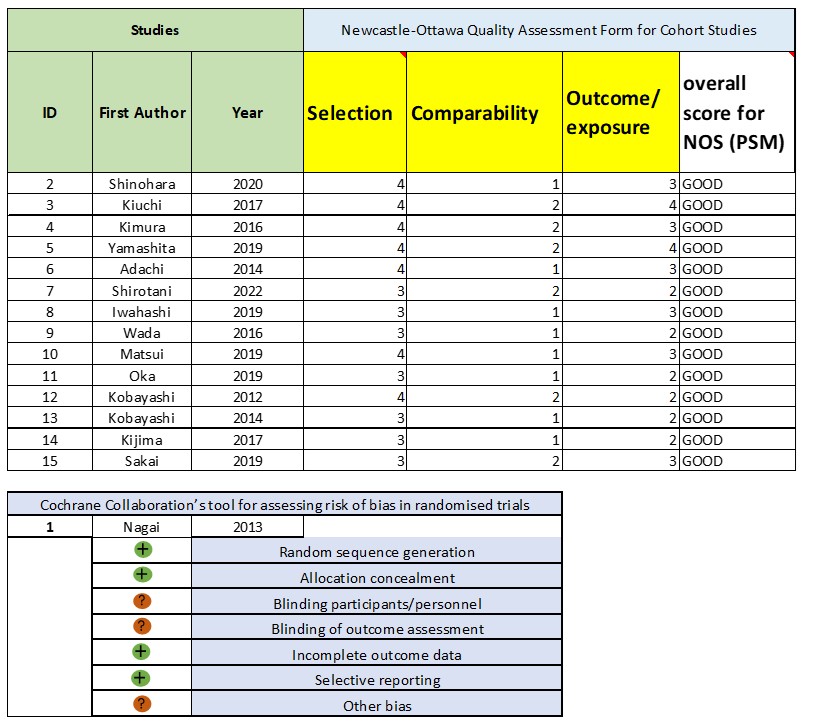

Supplement: Supplementary file 1 [file jcm-13-01683-s001.zip › Figure S1 Quality assessment.jpg]
